# Dermoscopy-based Radiomics Help Distinguish Basal Cell Carcinoma and Actinic Keratosis: A Large-scale Real-world Study Based on a 207-combination Machine Learning Computational Framework

**DOI:** 10.7150/jca.94759

**Published:** 2024-04-23

**Authors:** Hewen Guan, Qihang Yuan, Kejia Lv, Yushuo Qi, Yuankuan Jiang, Shumeng Zhang, Dong Miao, Zhiyi Wang, Jingrong Lin

**Affiliations:** 1 Department of Dermatology, First Affiliated Hospital of Dalian Medical University, Dalian, Liaoning, China.; 2 Department of General Surgery, First Affiliated Hospital of Dalian Medical University, Dalian, Liaoning, China.

**Keywords:** machine learning, basal cell carcinoma, actinic keratosis, dermoscopy, artificial intelligence

## Abstract

This study has used machine learning algorithms to develop a predictive model for differentiating between dermoscopic images of basal cell carcinoma (BCC) and actinic keratosis (AK). We compiled a total of 904 dermoscopic images from two sources — the public dataset (HAM10000) and our proprietary dataset from the First Affiliated Hospital of Dalian Medical University (DAYISET 1) — and subsequently categorised these images into four distinct cohorts. The study developed a deep learning model for quantitative analysis of image features and integrated 15 machine learning algorithms, generating 207 algorithmic combinations through random combinations and cross-validation. The final predictive model, formed by integrating XGBoost with Lasso regression, exhibited effective performance in the differential diagnosis of BCC and AK. The model demonstrated high sensitivity in the training set and maintained stable performance in three validation sets. The area under the curve (AUC) value reached 1.000 in the training set and an average of 0.695 in the validation sets. The study concludes that the constructed discriminative diagnostic model based on machine learning algorithms has excellent predictive capabilities that could enhance clinical decision-making efficiency, reduce unnecessary biopsies, and provide valuable guidance for further treatment.

## 1. Introduction

Basal cell carcinoma (BCC) is one of the most common types of malignant skin tumours, and its incidence is increasing annually 1. Diagnosed cases of BCC were approximately 3.6 million in 2022 2. As this type of skin cancer is more common in individuals over 50 with fair skin, population aging is one of the main risk factors 3, and exposure to ultraviolet radiation is one of the common etiological factors 4. In the United States, more than two million people are affected annually, solidifying BCC's position as one of the most widespread cancers among Caucasians 5, 6. In the United States, the annual growth rate of treatment costs for non-melanoma skin cancers, including BCC, has exceeded that of all other cancers. This has resulted in a significant burden on healthcare economics 7, 8.

Although BCC is known for its relatively low malignancy, its local invasiveness has the potential to disrupt surrounding tissues and organs. High-risk BCC may even pose a threat to a patient's life. Consequently, early intervention and treatment are essential 9, 10. Currently, due to the absence of specific molecular markers for diagnosis, the primary method for screening BCC involves clinical observation by dermatologists combined with dermoscopic examination. This approach offers the advantages of non-invasiveness and cost-effectiveness 11. However, for patients with BCC exhibiting atypical skin dermoscopic images, clinical challenges often arise, potentially resulting in underdiagnosis or misdiagnosis. Consequently, there is a risk of missing the optimal period for early intervention and treatment, presenting a significant diagnostic and therapeutic challenge 12. In the treatment guidelines for BCC, surgery stands out as the foremost approach for all types of BCC. Patients who receive early treatment typically also receive a more favourable prognosis, with a significantly reduced likelihood of complications occurring 13. It is noteworthy that the dermoscopic features of BCC closely correlate with its histological phenotype. For example, blue-grey ovoid nests are less common in superficial BCC compared with other subtypes 14. The presence of maple-leaf-like areas with branching vessels and ulcers often predicts a tendency toward the superficial subtype of BCC 15, 16. Moreover, studies have indicated that dermoscopic examination exhibits lower sensitivity and specificity in diagnosing non-pigmented BCC compared with pigmented BCC 17. Atypical dermoscopic features of BCC significantly overlap those of benign skin tumours, posing a challenge in the differential diagnosis between BCC and benign tumours 18. Unfortunately, typical dermoscopic features are observed in only a fraction of all BCC patients. The rest often necessitate a skin biopsy to confirm the diagnosis 19. To prevent unnecessary biopsies and surgeries, it is crucial to develop a discriminative diagnostic model with image-processing capabilities.

In routine dermatological practice, actinic keratosis (AK) is a condition often confused with BCC because some of AK's morphological features closely resemble BCC. Research suggests that differential diagnostic methods are crucial in diagnosing BCC involving entities such as AK and inflammatory skin diseases 14. In recent years, the incidence rate of AK has been steadily increasing in the population, making it a significant component in the differential diagnosis of BCC 20. AK is a prevalent intraepithelial tumour originating from keratinocytes, and it stands as one of the most frequently diagnosed diseases by dermatologists. Both BCC and AK are associated with prolonged exposure to ultraviolet radiation, and they tend to occur most frequently in sun-exposed areas of the face 21. Current research has confirmed that early interventions for AK can slow its progression, reduce the occurrence of complications, and lower the likelihood of transformation into invasive squamous cell carcinoma. In contrast to BCC, which is preferably treated surgically, the treatment for AK typically involves topical medication and physical therapies 22. Due to the highly similar affected sites of BCC and AK but markedly different treatment approaches, distinguishing between them in atypical presentations or early stages is challenging yet necessary 23, 24.

Dermoscopy, as an auxiliary diagnostic tool, has increased the accuracy of diagnosing various skin conditions. The availability of dermoscopy equipment in most hospitals has made dermoscopic images easily accessible in dermatological diagnosis and treatment 25. The current method of relying on a limited number of dermatologists to manually screen dermoscopic images for disease diagnosis is time-consuming, labour-intensive, and vulnerable to the subjective influence of doctors. Unfortunately, the rapid popularisation of dermoscopy as an auxiliary diagnostic tool has led to a relative shortage of doctors with rich dermoscopic experience. In summary, the accuracy of dermoscopic diagnosis faces significant challenges.

Deep learning (DL) neural networks exemplify the successful integration of artificial intelligence's automated processing into clinical practice, showcasing outstanding performance in tasks like image processing and classification, including applications in ultrasound and CT (computerised tomography) 26-28. Research indicates that the classification ability of deep neural networks for dermoscopic images can rival that of dermatologists 29. In recent years, machine learning has been extensively applied to structured data in genomics — particularly cancer genomics, where it is employed to identify and analyse pathogenic mutations. 28, 30, 31. Machine learning algorithms, with the aid of automated computer analysis, can learn from input data and optimise algorithmic combinations along with their parameters. This method is frequently employed in constructing prognosis and diagnostic models. In the realm of dermoscopic image classification, the application of this method has yielded substantial results 32. However, the successful implementation of these methods necessitates a substantial number of dermoscopic images for training. To overcome this challenge, this study integrated a significant amount of dermoscopic data from public and hospital databases to construct a highly discriminative diagnostic model for BCC and AK. We conducted comprehensive validation of the model. Currently, research on the classification of dermoscopic images based on DL models is primarily concentrated on the discrimination and diagnosis of melanoma, with limited application to the discrimination and diagnosis of BCC.

This study aimed to develop and evaluate a machine-learning-based model for the automatic discernment of BCC and AK from skin images. We believed that by combining advanced image-processing techniques with various machine learning algorithms, we could build a faster and more accurate discriminative diagnostic model for clinicians, with the potential to play a proactive role in managing early skin cancers.

## 2. Materials and Methods

### 2.1 Data Collection and Processing

We began by gathering and organising skin dermoscopic images from two datasets for subsequent analysis: the International Skin Imaging Collaboration (ISIC) dataset, a publicly accessible dataset used for skin disease diagnosis and research, and a dataset from the First Affiliated Hospital of Dalian Medical University.

From the ISIC open database, we selected the HAM10000 dataset, comprising 10,015 skin dermoscopic images and widely used in academic research. We downloaded and curated 514 BCC and 327 AK cases of skin dermoscopic images from this dataset, all diagnosed through histopathology.

For this retrospective study, skin dermoscopic images from the First Affiliated Hospital of Dalian Medical University were collected from February 2021 to October 2023. Our study was approved by the university's Ethics Committee and adhered to the principles of the Helsinki Declaration (approval no. PJ-KS-KY-2024-94). We obtained written informed consent for the dermoscopic images. After excluding images of low quality or with artifacts, we had 32 skin dermoscopic images of BCC and 31 of AK (DAYISET 1). All BCC cases underwent diagnosis through histopathology. For AK patients, diagnosis was conducted by two dermatologists with over ten years of dermoscopy experience in considering clinical symptoms. Images with a consensus diagnosis were included in the study. DAYISET 1 utilised the FotoFinder dermoscope. Subsequently, we performed a statistical analysis of the clinical data, including age, gender, and affected areas for each patient.

### 2.2 Data Segmentation

Similar to previous approaches 33, we used the “caret” package in R for random grouping to assess the performance of our developed diagnostic model. This package divides the data into approximately a 1:1 ratio, allocating 50% of HAM10000 as the training set (Dataset A) (n=422) and the remaining as internal test set 1 (Dataset B) (n=419), with all images serving as internal test set 2 (Dataset C) (n=841). To validate the model's accuracy and enhance clinical applicability, we conducted validation on our predictive model using DAYIDET 1. Dermoscopic images from DAYISET 1 were employed as an external validation set (Dataset D) (n=63).

### 2.3 Image Pre-processing and Deep Learning Feature Extraction

We pre-processed the images by resizing the pixel dimensions to 600×450. Leveraging transfer learning techniques, we then applied the resnet50 model for pre-training on our images. We fine-tuned the learning parameters based on the optimal model output from the pre-training results. Subsequently, we retrained the model and extracted deep-learning features with dimensions of 2048 through the average pooling layer. The obtained feature values were normalised to the range of 0-1 for subsequent transfer learning. Due to the limited interpretability of DL models, we applied Gradient-weighted Class Activation Mapping (Grad-CAM) for the visualisation of our input images and to gain further insights into the decision-making process of our developed model. This method highlights the input image regions crucial to the model's decision and presents them through a heatmap.

### 2.4 Feature Selection

To improve the efficiency of subsequent analysis and avoid overfitting, we utilised the Limma package in R to perform differential analysis on DL features. We selected the top 30 differentially expressed features with the smallest p-values between groups for further modelling analysis. The p-values for the features selected for subsequent modelling were all less than 0.05, indicating significant statistical differences.

### 2.5 Constructing a Predictive Model through Machine Learning Algorithms

Machine learning algorithms, through leveraging large datasets and advanced algorithms, possess powerful data analysis capabilities. They are increasingly being applied to the field of medicine, demonstrating excellent performance in many studies 34-36. To enhance the accuracy and stability of our newly developed diagnostic model, and to expedite the efficiency of image automation in personalised medicine, we chose the best model through machine learning algorithms to achieve optimal diagnostic performance. Firstly, these machine learning algorithms encompass 15 different types of classical algorithms, specifically including neural networks, logistic regression, linear discriminant analysis (LDA), quadratic discriminant analysis (QDA), K-nearest neighbours (KNN), decision tree, random forest (RF), XGBoost, ridge regression, least absolute shrinkage and selection operator (LASSO), elastic net regression, support vector machine (SVM), gradient boosting machine (GBM), stepwise logistic regression, and naive Bayes. We combined hyperparameter tuning, custom parameter combinations, Lasso feature selection, and cross-validation using the caret package, resulting in a total of 207 unique machine learning models. For different models, we compared the performance of various classifiers by calculating the receiver operating characteristic (ROC) curve to assess the diagnostic efficacy of the models. We employed three commonly used machine learning metrics — F-score, accuracy (ACC), and recall — to evaluate model performance. By considering these statistical indicators collectively, we selected the optimal model and its parameters. Currently, there is limited research on machine learning for binary classification problems in the dermatoscopic field. We have pioneered the development of a composite model obtained through machine learning algorithms and used cross-validation for model training and evaluation.

### 2.6 Validation of the Diagnostic Model

Based on the outcomes of our prior data segmentation and the optimal parameters derived from machine learning algorithms, we generated ROC curves and calibration curves for distinct datasets, including Dataset A, Dataset B, Dataset C, and Dataset D. Moreover, we computed the area under the curve (AUC) values to assess and compare the diagnostic accuracy of the models across these diverse datasets.

To further validate the efficacy of our developed machine learning model for binary classification, we scrutinised the actual diagnostic outcomes against the model's predictions using a confusion matrix. The visualisation of this matrix was accomplished using the Matplotlib and Seaborn packages in Python. Matplotlib was employed to construct the graphical interface, while Seaborn contributed to enhancing the visual appeal of the graphics and streamlining the plotting of the confusion matrix. Additionally, the confusion-matrix function from the Sklearn package was used to compute the confusion matrix, a widely used tool for evaluating the predictive performance of classification models.

### 2.7 Statistical Analysis

Our study utilised Python (version 3.9) and R language (version 4.3.0) for image processing, data analysis, and visualisation operations. For the evaluation of binary classification model performance, we employed confusion matrices and ROC curves to quantify the model's classification accuracy and performance. For the statistical analysis of baseline data, we used SPSS 26.0 software. P-value<0.05 was considered statistically significant.

## 3. Results

### 3.1 Workflow of the Study

Firstly, our study included patients from the HAM10000 and DAYISET 1 databases, divided into four cohorts using a random grouping method. Dermoscopic images of their lesion sites were collected using a dermatoscope. Similar to previous studies, the significance threshold for differential analysis was set at 0.05, with a fold change of 1 37. The results of the differential analysis were visualised using a volcano plot (Supplementary Figure S1).

**Figure 1:**
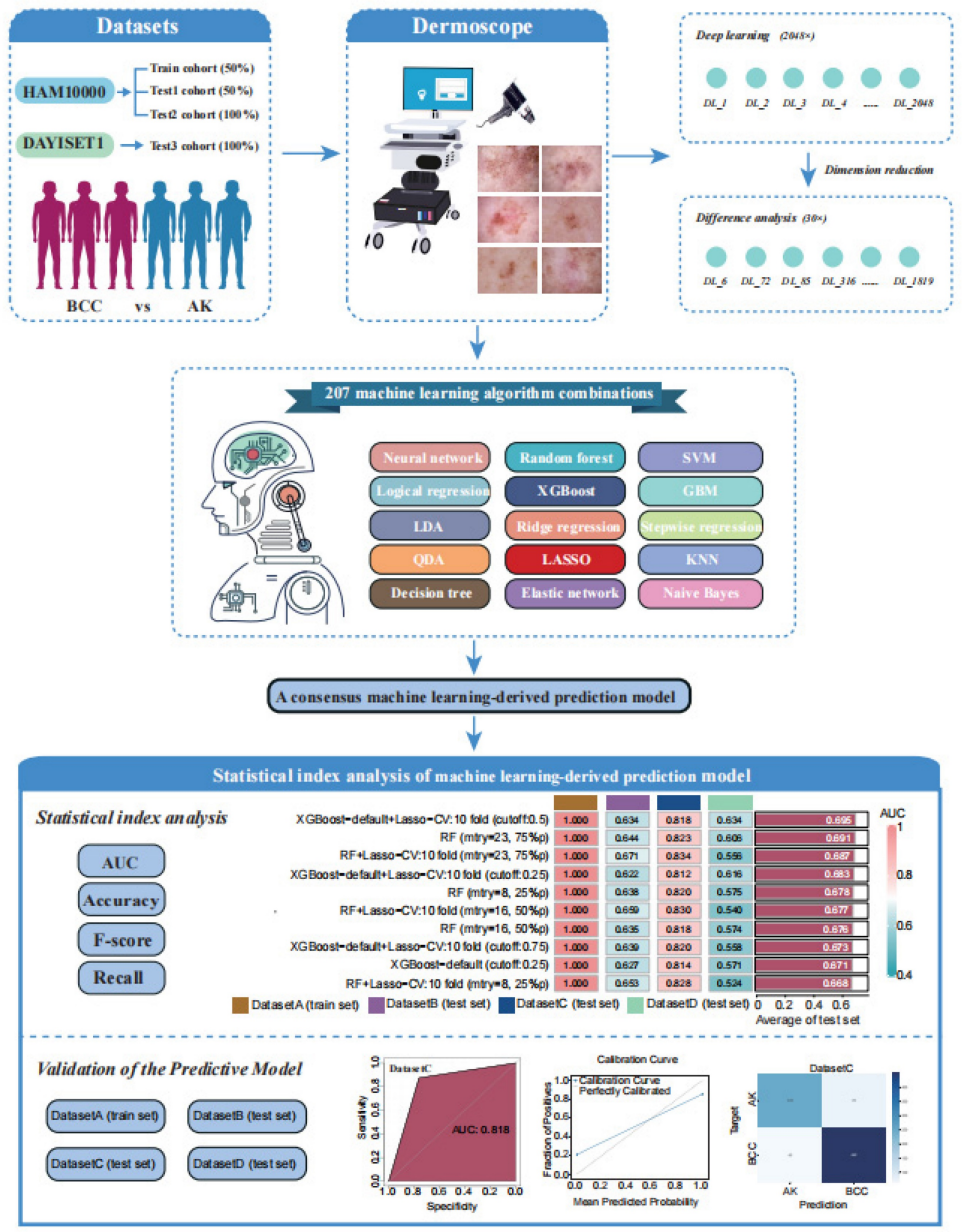
The workflow of our machine learning prediction model development.

**Figure 2:**
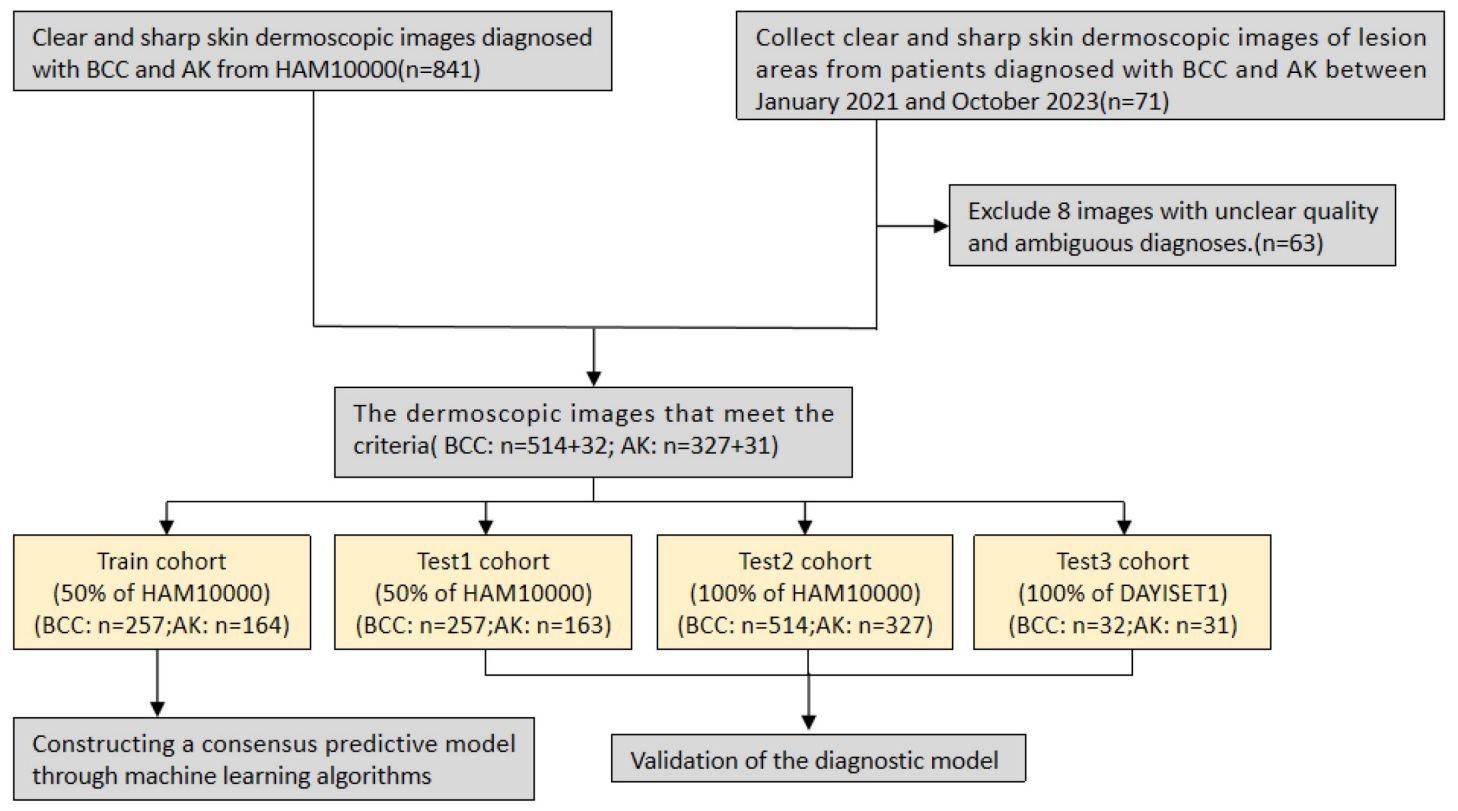
Flowchart of study inclusion.

Next, leveraging a machine learning framework, we integrated 15 machine learning algorithms and conducted model training and parameter optimisation through cross-validation and threshold adjustment for the binary classification model. Subsequently, we computed and ranked the AUC values, accuracy, and F-score of different combined models in each cohort, resulting in a consistently performing machine learning model. To validate the predictive capacity of this model, we visualised its calibration curve and confusion matrix. The research workflow is depicted in Figure 1. The flowchart provides detailed information on inclusion/exclusion criteria and grouping methods for our dermoscopic images (Figure 2).

### 3.2 Patients and Clinical Features

Table 1 summarises the demographic data and lesion locations for the two cohorts. A total of 904 patients were included in the training and testing sets, originating from HAM10000 (with BCC accounting for 61.1%, 514/841) and DAYISET 1 (with BCC accounting for 50.8%, 32/63). There were 546 patients with BCC, with an age of 66.83±13.66 years in the HAM10000 dataset and 69.84±10.18 years in the DAYISET 1 dataset. The male proportion was 61.7% in HAM10000 and 40.6% in DAYISET 1. Additionally, there were 358 patients with AK, with an age of 66.53±11.48 years in HAM10000 and 70.06±11.98 years in DAYISET 1. The male proportion was 67.5% in HAM10000 and 38.7% in DAYISET 1. The predominant affected areas for both diseases were the head and face, accounting for 162/546 in BCC patients and 171/358 in AK patients.

### 3.3 Visualisation Results of Grad-CAM

**Figure 3:**
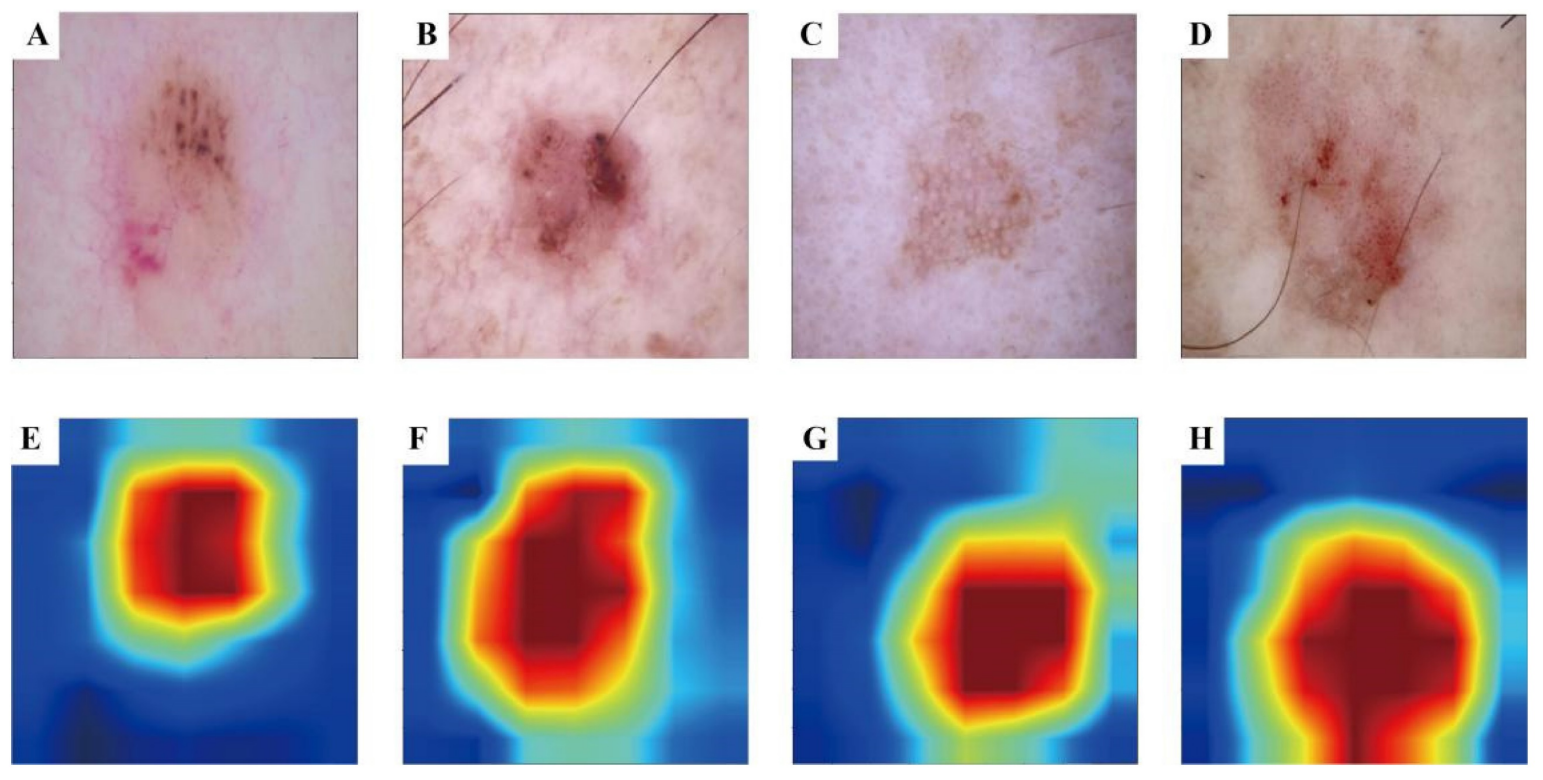
Grad-CAM visualisation for BCC and AK classification model. The top row is the original dermatoscope image, and the bottom row is the Grad-CAM result image.

To enhance the interpretability of our advanced DL model and illuminate the decision-making process, we used the Grad-CAM technique. This method generates heatmaps to accentuate the input image regions pivotal to the model's decision. Figure 3 A-D shows the original images during prediction, whereas Figure 3 E-H illustrates the heatmaps created using Grad-CAM. In these heatmaps, the red region indicates the area most prioritised by the model in recognition, succeeded by yellow, green, and blue areas.

### 3.4 Development of Machine Learning Prediction Model

**Figure 4:**
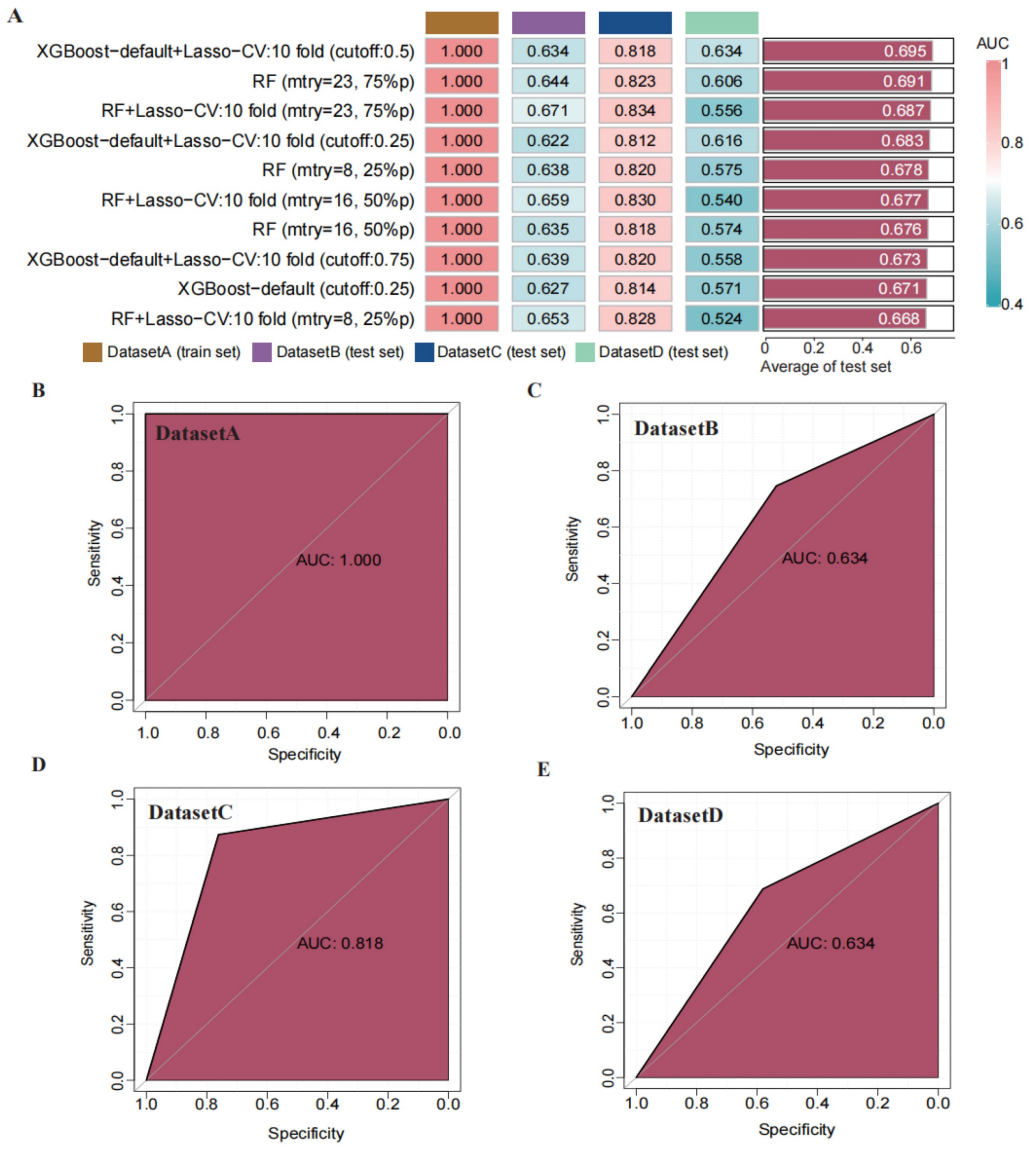
Construction and validation of a consensus machine learning diagnosis model. The top 10 among 207 machine learning prediction models are developed through ten-fold cross-validation based on 15 classical algorithms, and the AUC values of each model are calculated. (B) ROC curve of the predictive model in the training set cohort. (C) ROC curve of the predictive model in the internal validation set 1 cohort. (D) ROC curve of the predictive model in the internal validation set 2 cohort. (E) ROC curve of the predictive model in the external validation set cohort.

Through transfer learning, we inputted the top 30 differentially significant deep learning features (obtained through differential analysis) into machine learning algorithms for training. Using 207 machine learning algorithms, we fine-tuned and trained the models on the training set, calculated the mean AUC values across the three validation sets, and ranked the models to evaluate their predictive capabilities (Figure 4 A). The rankings of AUC, diagnostic accuracy, and F-score calculations for our model are found in the supplementary materials Figure S3. Based on the training results, we established a consensus model, which was built on the default configuration of XGBoost combined with Lasso regression. This model underwent training through 10-fold cross-validation, with a threshold set at 0.5 for the binary classification task. Subsequently, we plotted the ROC curves of this model in different datasets and calculated the AUC values, which were 1.000 in the training set, 0.634 in DATASET 2, 0.818 in DATASET 3, and 0.634 in DATASET 4.

### 3.5 Validation and Performance Assessment of the Predictive Model

**Figure 5:**
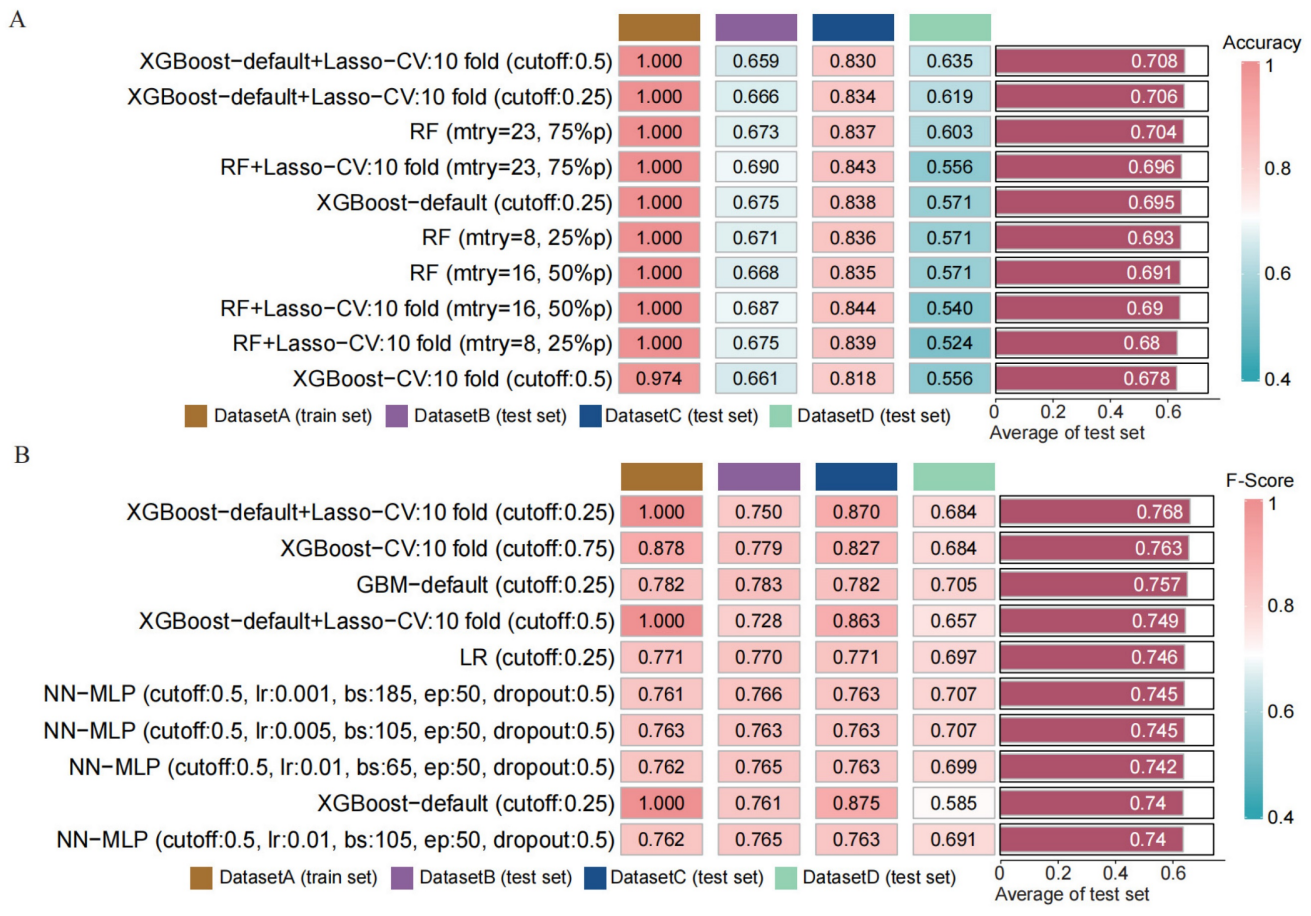
Comparison of the performance of machine learning prediction models in different data sets and calculation of the mean in the test set. (A) Calculation of diagnostic accuracy of models in different datasets. (B) Calculation of F-score of models in different datasets.

**Figure 6:**
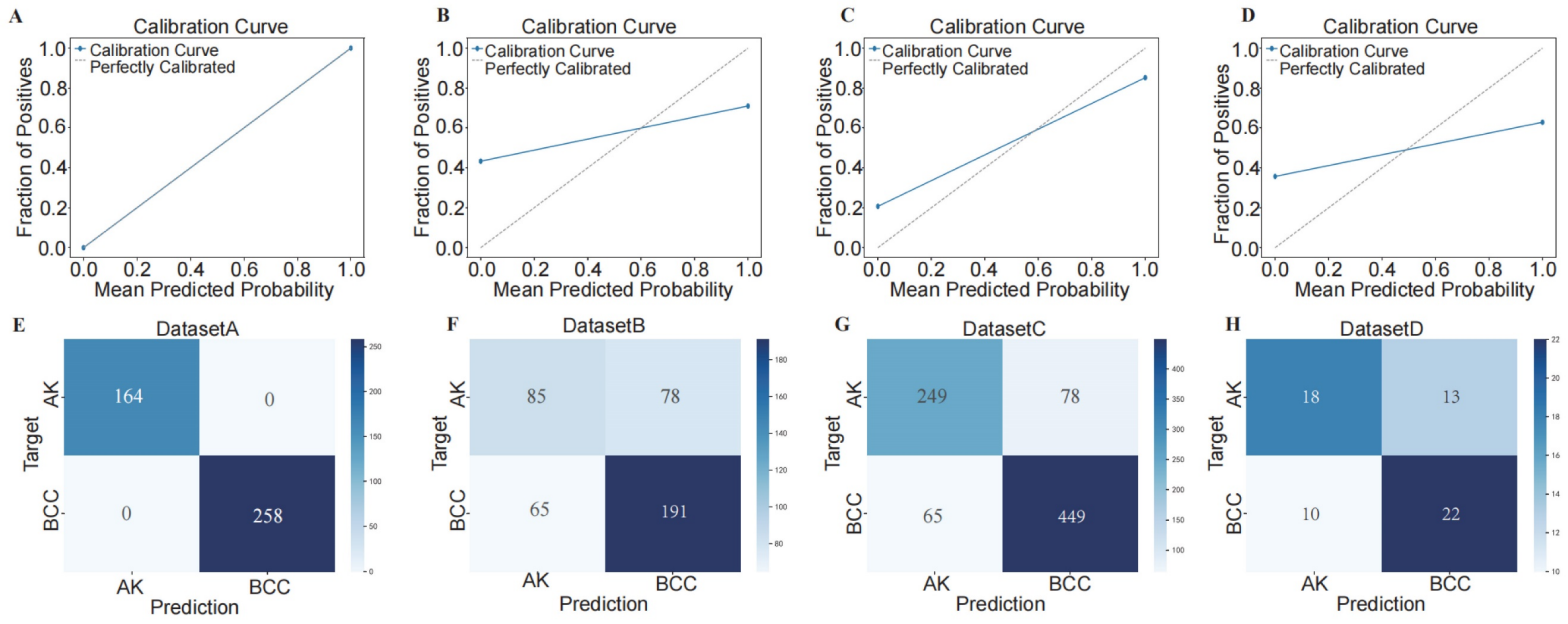
Using confusion matrix and calibration curve, the predicted results of this consensus diagnostic model compared with the actual results. (A-D) Calibration curves of training set, internal verification set 1, internal verification set 2 and external verification set. (E-H) Confusion matrix of training set, internal verification set 1, internal verification set 2 and external verification set.

To assess the performance of our developed classification model, we computed common binary classification metrics, including accuracy (Figure 5 A), F-score (Figure 5 B), and recall Figure S2. In the training set, the model achieved perfect values for accuracy, F-score, and recall, all equal to 1.000. In the validation set, the mean values were 0.708 for accuracy, 0.768 for F-score, and 1.000 for recall. To illustrate the extent of probability drift in our model, we plotted calibration curves for different datasets (Figure 6 A-D). The horizontal axis of the calibration curve represents the predicted probabilities, and the vertical axis represents the actual probabilities. In the training set, our calibration curve perfectly overlapped with the ideal calibration curve. In the test sets, the calibration curve for test set 2 showed the smallest deviation angle.

Next, we illustrated the model's classification results more clearly by plotting confusion matrices. The confusion matrices are depicted in Figure 6 E-H, where rows represent the actual categories, and columns represent the model's predicted categories. Using the confusion matrices, we computed the sensitivity for diagnosing BCC in different datasets. The sensitivity for DATASET 1 was 1.0, DATASET 2 was 0.746, DATASET 3 was 0.874, and DATASET 4 was 0.688.

## 4. Discussion

BCC is a common non-melanoma skin tumour, and its specific manifestations in appearance vary depending on the degree of infiltration. Therefore, some skin lesions can also exhibit dermoscopic features similar to BCC. This characteristic leads to difficulty in distinguishing skin lesions, requiring confirmation through biopsy after histopathological examination. For example, certain squamous cell carcinomas can present a basaloid appearance 38, and the abnormal dermoscopic pattern of BCC is like that of melanoma 39. The mainstay of treatment for BCC is surgical excision. However, in the case of diseases that can be misidentified as BCC, such as AK, surgical intervention is often the non-preferred option. The precision and effectiveness of non-invasive dermoscopic examinations for preoperative BCC diagnosis, particularly in cases lacking typical dermoscopic features, still fall short. Hence, enhancing the preoperative dermoscopic detection rate of BCC is of paramount importance. With the advent of artificial intelligence and big data, an increasing number of image-recognition assessment models tailored to medical imaging data are being applied to clinical tasks that challenge discernment. Radiomics has demonstrated excellent performance in recognition and classification tasks involving medical images, encompassing ultrasound, CT scans, endoscopy, and dermatoscopy 40-43. In the field of dermatoscopy, there is a growing body of research on constructing classification models based on deep learning. However, most studies analyse dermatoscopic images from publicly available datasets. Our research integrates imaging data from public databases as a training set, establishes an internal validation set through data partitioning, and uses dermatoscopic images from our hospital as an external validation set. The classification model, validated through multiple datasets, exhibits enhanced diagnostic performance and stability across validation sets.

Artificial intelligence often demonstrates excellent processing capabilities in image processing and can simulate the human learning process through computer algorithms. In the medical field, artificial intelligence can be utilised for the analysis and diagnosis of medical images, such as detecting tumours and identifying signs of disease 44-46. In the field of skin disease diagnosis, a recent study has utilised reflectance confocal microscopy for the differential diagnosis of skin diseases, and it has shown promising performance. In a recent study, this approach was employed to develop a diagnostic model for BCC 47. In practical applications, this imaging acquisition method is less commonly used compared with dermoscopy devices, leading to greater difficulty in image acquisition and a lack of validation through extensive publicly available data. However, the performance of machine learning models depends heavily on the quality and quantity of their training data. Machine learning models have numerous parameters, and with a substantial number of samples, the model can better adjust these parameters to accommodate the complexity of the training data. This helps the model capture abstract features in the input data more effectively and mitigates overfitting, making the model more generalisable 48. Our study enhances the universality and stability of the classification model through extensive computer training using a large volume of dermoscopic images.

Recently, significant progress has been made in the identification of molecular biomarkers and the construction of prognostic models using machine learning models. For instance, in the study conducted by Zhang et al. 49, a combination model trained through machine learning can more precisely identify glioma patients who may benefit from immunotherapy, thereby enabling personalised medical treatment for these patients. In current research, the combinations of machine learning model algorithms are often generated by randomly combining common algorithms, typically around ten in number 30. In our study, however, we employed a random combination and parameter tuning process with 15 classical algorithms, resulting in a total of 207 algorithmic models. With the increase in the number of trained models, the computational workload for the computer learning tasks has significantly increased. Instead of a simple permutation and combination of algorithms, we applied methods like cross-validation to compute the machine learning with different parameters, obtaining optimal model parameters. In the field of dermoscopy DL models, many studies construct models using classical single algorithms, such as Lasso and GBM 50-52. Contrary to previous research methods, our study introduced innovation in model construction by leveraging machine learning algorithms. The resultant models demonstrate a notable superiority over traditional counterparts, effectively addressing the limitations of conventional modelling approaches.

In conclusion, we've created a composite algorithmic model that incorporates deep transfer learning (DTL) features using machine learning to differentiate between BCC and AK, validated across various cohorts. This composite model consistently surpasses the performance of individual classical models. Moreover, the constructed DTL model demonstrates notable specificity and sensitivity, assisting physicians of diverse experience levels in improving diagnostic accuracy and efficiency.

In this retrospective study, we utilised both public databases and dermoscopic images from our medical centre to construct and validate a novel predictive model for distinguishing between BCC and AK. The DL model employed a pre-training approach combined with machine learning modelling, using high-throughput automated feature extraction algorithms to extract features from images and transform them into quantitative features 53. In this research, we employed a pre-trained DL model to process images, extracting features that were then transformed into quantitative data for subsequent analysis. The DTL technique facilitated the transfer of features between domains through feature transformation, aiming to minimise the feature gap between the source and target domains. Developed on DL algorithms, this technique is designed to improve the model's generalisation 54. DTL automates the processing of input images, conducts target segmentation through weak supervision, and extracts DTL features. Employing computer-based machine learning model training, the model generated by combining the default configuration of XGBoost with Lasso regression showed superior predictive performance in both training and testing datasets compared with other models. The average AUC across the four datasets was 0.695, with a training set AUC value of 1.0. Furthermore, our model exhibited substantial sensitivity and specificity. In detail, when comparing the model's predictions to the actual outcomes, the sensitivity was 1.0 in the training set and 0.746, 0.874, and 0.688 in the remaining three validation sets.

In summary, our model exhibits consistent predictive performance, providing a non-invasive approach for preoperative patient assessment. Additionally, we transformed the extracted quantitative feature values into heatmaps using Grad-CAM. Upon reviewing these heatmaps, we observed that, for dermoscopic images where the lesion area was not explicitly outlined, our model autonomously identified and extracted the lesion area, effectively delineating the boundary between the tumour and normal skin tissue. This approach serves to confirm the robustness of our model in image recognition.

Conclusively, dermoscopy stands out as a straightforward tool for distinguishing between BCC and AK, boasting the benefits of non-invasiveness and cost-effectiveness. Improving the discriminative performance and efficiency of this supplemental examination in skin disease diagnosis holds considerable clinical significance and has the potential for broad integration into standard dermatological practices. The machine learning model we devised showcased outstanding predictive abilities across various validations. This decision support system has the potential to function as a supplementary diagnostic tool in clinical environments and serve as a valuable learning aid for less experienced dermatologists and dermatology trainees.

However, our study has certain limitations, outlined as follows: 1 Our model was developed through a retrospective analysis of selected data, necessitating further prospective studies to validate the stability of our diagnostic model. 2 Due to the absence of subtype annotations for the lesions in our original data, our classification model cannot predict subtypes for BCC and AK. 3 Our research is based on the analysis and modelling of dermoscopic images from a public database and a single medical centre, and so lacks validation from large-scale, multicentre datasets.

## 5. Conclusion

In summary, our study is based on a combination model developed from 207 machine learning models, integrating XGBoost with Lasso regression. This model, utilising the automated processing of dermoscopic images, demonstrates exceptionally high accuracy in the discrimination and diagnosis of BCC and AK. The classification diagnostic model can assist less experienced dermatologists in distinguishing lesions, aiding in the selection of appropriate treatment strategies for patients and mitigating the need for biopsies.

## Supplementary Material

Supplementary figures.

## Figures and Tables

**Table 1:** Patient demographics and lesion location distributions of patients in different datasets

Characteristics	HAM10000			DAYISET 1		
	BCC	AK	p Value	BCC	AK	p Value
	(n=514)	(n=327)		(n=32)	(n=31)	
Age, mean±SD, year	66.83±13.66	66.53±11.48	0.742	69.84±10.18	70.06±11.98	0.937
Gender, n (%)			0.082			0.877
Male	317 (61.7)	221 (67.5)		13 (40.6)	12 (38.7)	
Female	197 (38.3)	106 (32.4)		19 (59.4)	19 (61.3)	
Location, n (%)			<0.001			0.234
Head and neck	134 (25.9)	140 (42.6)		28 (87.5)	31 (100)	
Face	101 (19.6)	113 (34.5)		24 (75.0)	30 (96.7)	
Scalp	19 (3.6)	14 (4.2)		4 (12.5)	1 (3.3)	
Ear	0 (0)	3 (0.9)		0 (0)	0 (0)	
Neck	14 (2.7)	10 (3.0)		0 (0)	0 (0)	
Upper extremity	49 (9.5)	62 (18.9)		2 (6.2)	0 (0)	
Hand	2 (0.3)	13 (3.9)		0 (0)	0 (0)	
Lower extremity	58 (11.9)	65 (19.8)		1 (3.1)	0 (0)	
Foot	4 (0.7)	0 (0)		0 (0)	0 (0)	
Back	186 (36.1)	29 (8.8)		1 (3.1)	0 (0)	
Trunk and abdomen	76 (14.7)	18 (5.4)		0 (0)	0 (0)	
Abdomen	18 (3.5)	5 (1.5)		0 (0)	0 (0)	
Chest	47 (9.1)	12 (3.6)		0 (0)	0 (0)	
Trunk	11 (2.1)	1 (0.3)		0 (0)	0 (0)	
Others	5 (0.9)	0 (0)		0 (0)	0 (0)	
