# Supplementary material for: Dermoscopy-based Radiomics Help Distinguish Basal Cell Carcinoma and Actinic Keratosis: A Large-scale Real-world Study Based on a 207-combination Machine Learning Computational Framework
Source: J Cancer. 2024 Apr 23;15(11):3350–61. doi: 10.7150/jca.94759 (PMC11134443; doi:10.7150/jca.94759)
Supplement: Supplementary file 1 — Supplementary figures. [file jcav15p3350s1.pdf]

Supplementary material for
Dermoscopy-based Radiomics Help Distinguish Basal Cell Carcinoma and Actinic Keratosis: A Large-scale Real-world Study Based on a 207-combination Machine Learning Computational Framework

Hewen Guan[1#], Qihang Yuan[2#], Kejia Lv[1], Yushuo Qi[1], Yuankuan Jiang[1], Shumeng Zhang[1],Dong Miao[1],Zhiyi Wang[1], Jingrong Lin[1*]

1. Department of Dermatology, First Affiliated Hospital of Dalian Medical University, Dalian, Liaoning, China.
2. Department of General Surgery, First Affiliated Hospital of Dalian Medical University, Dalian, Liaoning, China.

*Correspondence to: Jingrong Lin, E-mail: amy4963@163.com

Hewen Guan, Qihang Yuan contributed to this article equally.

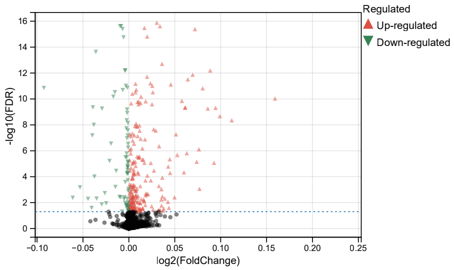

Figure S1 Visualization of the results of difference analysis.

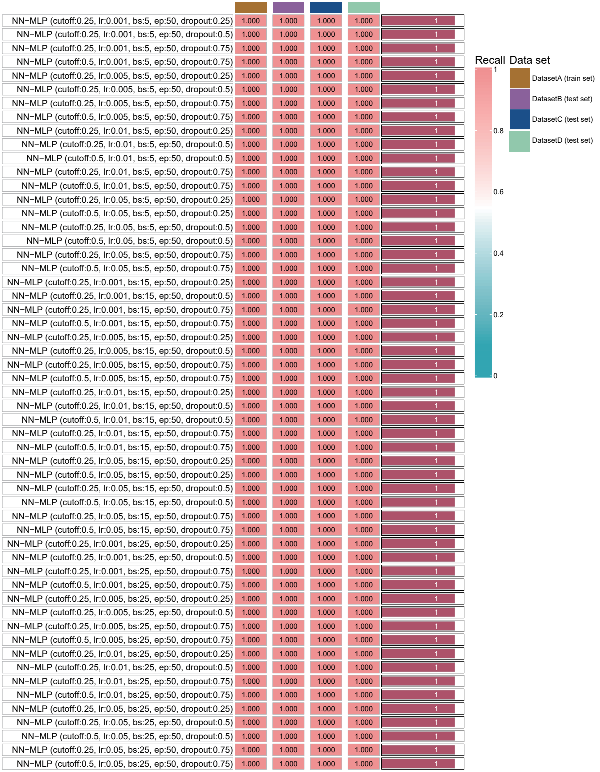

Figure S2 Recall rates of the machine learning models.

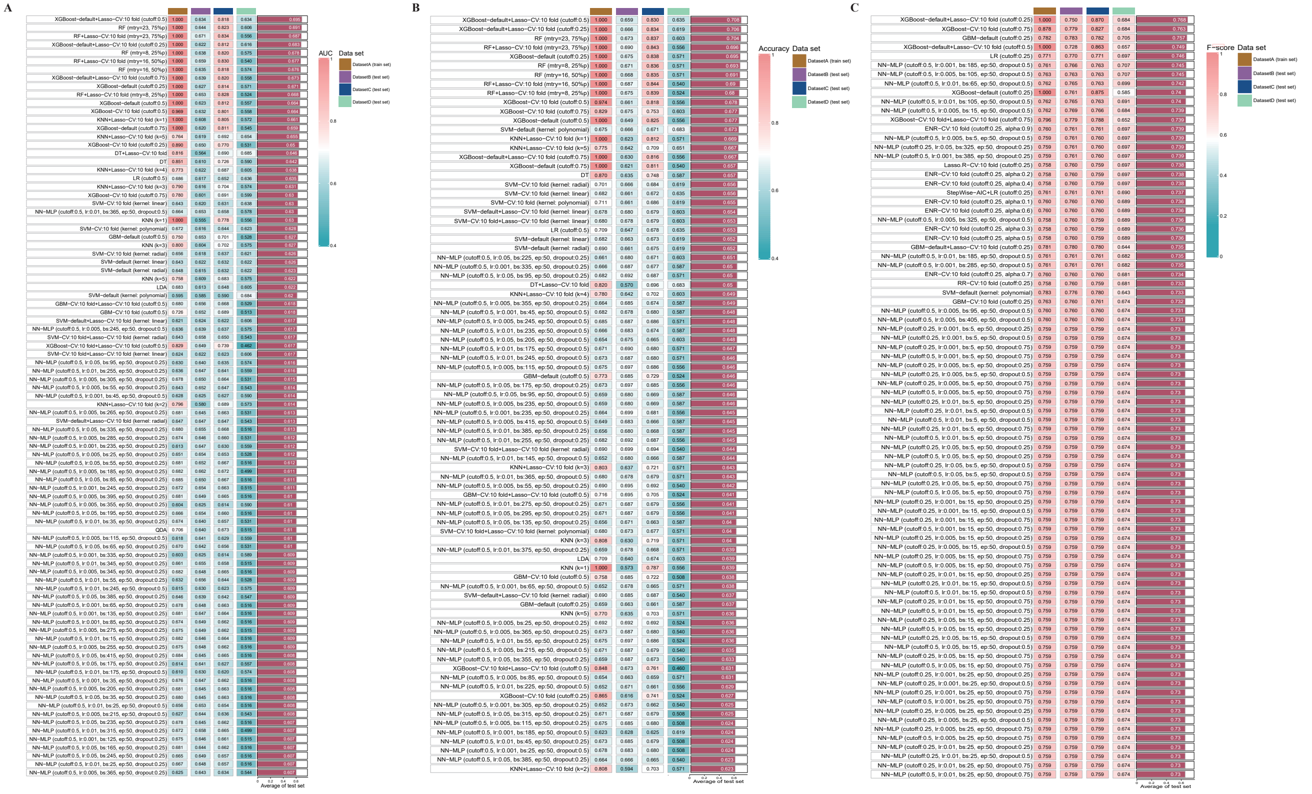

Figure S3 Compare the performance of machine learning prediction models in different data sets, and calculate the mean in the test set. (A)Calculation of AUC values of models in different datasets(B)Calculation of diagnostic accuracy of models in different datasets. (C) Calculation of F-score of models in different datasets.
